# The complex structure and function of Mediator

**DOI:** 10.1074/jbc.R117.794438

**Published:** 2017-09-14

**Authors:** Thomas M. Harper, Dylan J. Taatjes

**Affiliations:** From the Department of Biochemistry, University of Colorado, Boulder, Colorado 80303

**Keywords:** cryo-electron microscopy, gene expression, protein structure, transcription, X-ray crystallography, CDK8 module, Mediator, RNA polymerase II, cross-linking mass spectrometry

## Abstract

In eukaryotes, RNA polymerase II (pol II) transcribes all protein-coding genes and many noncoding RNAs. Whereas many factors contribute to the regulation of pol II activity, the Mediator complex is required for expression of most, if not all, pol II transcripts. Structural characterization of Mediator is challenging due to its large size (∼20 subunits in yeast and 26 subunits in humans) and conformational flexibility. However, recent studies have revealed structural details at higher resolution. Here, we summarize recent findings and place in context with previous results, highlighting regions within Mediator that are important for regulating its structure and function.

## Introduction

Regulation of RNA polymerase II (pol II)^2^ transcription in eukaryotes is carried out in many ways, from the DNA sequence and chromatin architecture to recruitment and regulation of large protein assemblies at the promoter (1, 2). Central to this regulation is the multisubunit Mediator complex, which appears to communicate regulatory inputs from DNA-binding transcription factors and promoter-bound complexes directly to the pol II enzyme. Mediator functions within the so-called preinitiation complex (PIC), which assembles at transcription start sites and regulates pol II recruitment and activity (3). The PIC contains Mediator, pol II, and the general transcription factors TFIIA, TFIIB, TFIID, TFIIE, TFIIF, and TFIIH (4–6). Although Mediator is generally conserved across eukaryotes, its sequences and subunit composition have diverged significantly (7). As shown in Table 1, in the yeast *Schizosaccharomyces pombe*, Mediator consists of 19 subunits and is ∼0.8 MDa in size, whereas in *Saccharomyces cerevisiae*, Mediator contains 21 subunits with a molecular mass of ∼0.9 MDa. By comparison, human Mediator contains 26 subunits (∼1.4 MDa), with five subunits (MED23, MED25, MED26, MED28, and MED30) that appear to be metazoan-specific.

**Table 1:**
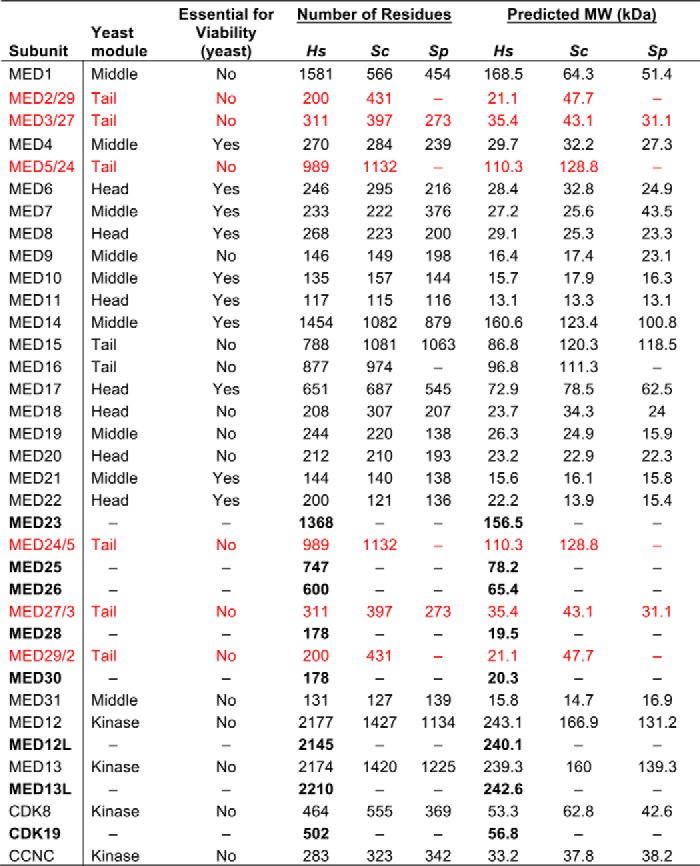
**Overview of Mediator subunits from *Homo sapiens* (*Hs*), *S. cerevisiae* (*Sc*), and *S. pombe* (*Sp*)** Subunits specific to humans are shown in bold font; subunits in red font were originally considered metazoan-specific (76) but later assigned orthologs in yeast (77).

Based upon initial two-dimensional projections of the complex, yeast Mediator has been divided into three structural modules called the head, middle, and tail (8). These designations required redefinition upon further study (9, 10), but they have conceptually guided biochemical reconstitution experiments that have greatly enhanced structural characterization of yeast Mediator complexes. Recent high-resolution structural data have shown how the head and middle modules interact with each other (11, 12). It remains to be established whether a similar structural architecture persists in human Mediator, but initial results suggest that the basic architectural framework is conserved (9, 13).

In addition to its large size, Mediator is structurally dynamic. Mediator subunits contain many intrinsically disordered regions (14), which have complicated its structural characterization. Moreover, a four-subunit kinase module (containing MED12, MED13, CDK8, and CCNC) can reversibly associate with Mediator, which adds to its structural and functional complexity. Whereas electron microscopy (EM) was initially able to establish basic structural features at low resolution (15–17), higher resolution data of the complex were lacking, until recently. Over the past 6 years, starting with work from Takagi and co-workers (18), the structural features of the Mediator complex have begun to come into focus (19). Most of the high/intermediate resolution data are derived from yeast Mediator (*S. pombe* or *S. cerevisiae*) through the use of X-ray crystallography, cryo-EM, and cross-linking mass spectrometry (CXMS). In this Minireview, we summarize the more recent structural studies and place them in context with previous findings. Throughout, we highlight regions within Mediator that are highly interconnected and/or appear to be especially important for regulating its structure and function.

## MED14, a backbone for Mediator

**Figure 1.**
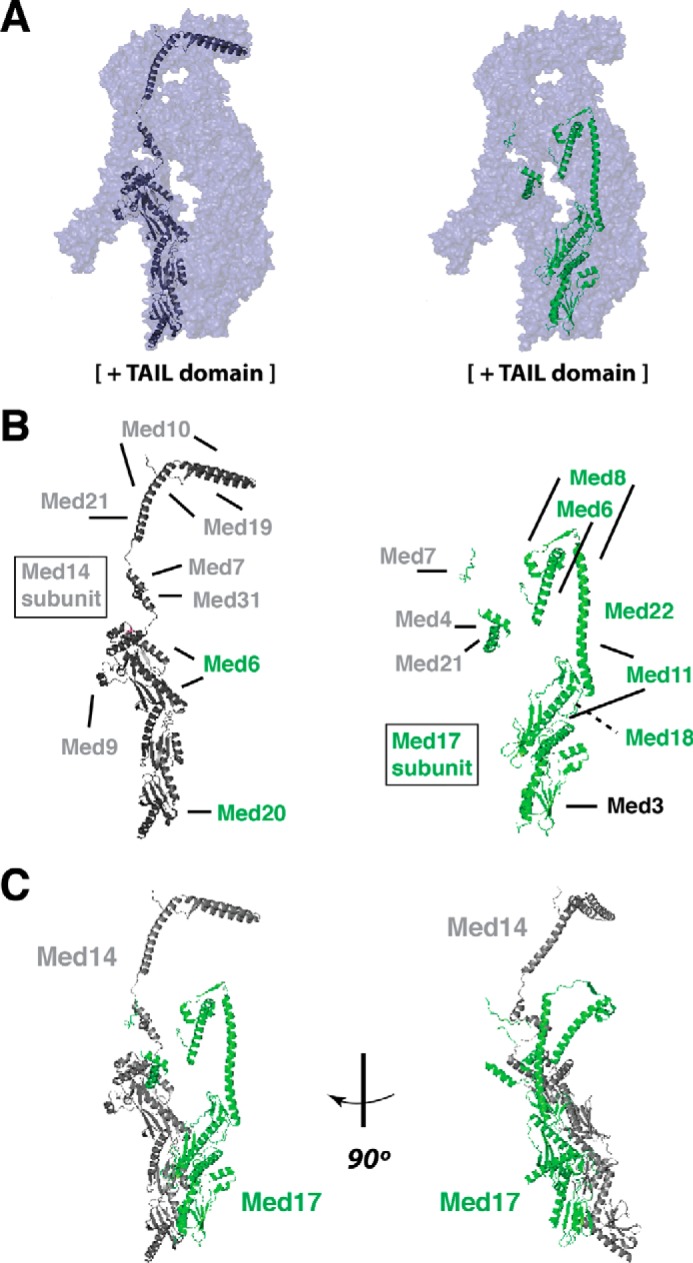
**Subunits Med14 and Med17 are Mediator structural hubs.** *A,* ribbon model for Med14 (*left*) and Med17 (*right*) shown super-imposed on the cryo-EM map of the “head + middle” Mediator structure (*S. pombe*). General location of tail domain (currently not resolved at high resolution) is indicated with text. *B,* summary of Med14 (*gray, left*) and Med17 (*green, right*) intersubunit contacts in Mediator, with a rough approximation of subunit proximity and interfaces, based upon structural data from Asturias and co-workers (12) and Cramer and co-workers (11). *Green font* corresponds to head module subunits, and *gray font* corresponds to middle module. Med3 is a tail module subunit. Note that Med14–Med17 interactions are not shown. *C,* Med14 and Med17 form extensive contacts, especially toward their C-terminal regions. Shown are ribbon models of the polypeptide backbone for Med14 (*gray*) and Med17 (*green*). Structures are from PDB 5N9J (11) and are constructed as shown in PyMOL.

In the yeast Mediator structure (*S. cerevisiae* or *S. pombe*), Med14 has been shown to play an essential role in holding the entire assembly together. Med14 makes contacts with all three modules of Mediator (head, middle, and tail) and serves as a backbone (Fig. 1) for the entire complex (9, 11, 12, 20, 21). Med14 interacts with numerous middle module subunits and contacts Med17, Med6, and Med20 in the head module (Fig. 1*B*). The Med17–Med14 interface is extensive (∼3000 Å^2^, Fig. 1*C*), and the Med14–Med20 contact appears crucial to stabilize the head orientation. Med14 also appears to contact the tail module subunits Med2 and Med15 through its C terminus (12, 21, 22). As a highly interconnected backbone, Med14 may help direct structural changes throughout the Mediator complex (see below). The structural architecture of yeast Med14 appears to be conserved in the human Mediator complex (9). By reconstituting a sizable portion of the human Mediator complex (15 subunits), Roeder and co-workers (13) showed MED14 cross-linked to subunits assigned to either the head or middle modules, and MED14 was able to biochemically associate with head, middle, or tail subunits.

## MED17 as structural hub

A key structural role for Med17 in yeast Mediator was inferred through genetic studies that identified a Med17 temperature-sensitive mutant strain (srb4-138) that de-stabilized the entire complex at the nonpermissive temperature (23, 24). Med17 comprises the largest contact between the head and the rest of the Mediator structure (9) and appears to form an extensive head–tail interface that mostly involves contacts with the C-terminal region of Med14 (11, 12). CXMS and structural data reveal an extensive set of intersubunit interactions for Med17 (Fig. 1*B*) that include head module subunits Med6, Med8, Med11, Med18, and Med22 (20–22, 25). In addition, the N terminus of Med17 helps tether the head and middle modules through interactions with Med4, Med14, and Med7C and Med21 (11, 12, 21). Perhaps most striking about Med17 is its extensive set of contacts with Med14 (11, 12), the “backbone” of yeast Mediator that connects head, middle, and tail modules (Fig. 1*C*). Consistent with the role of MED17 as a structural hub, Roeder and co-workers (13), through reconstitution of a 15-subunit “core” human Mediator assembly, identified an extensive set of cross-links between MED17 (especially toward the N-terminal portion of the 651-residue MED17 protein) that included MED6, MED8, MED11, MED22, and the metazoan-specific subunit MED30.

## Arm, spine, hook, and knob

**Figure 2.**
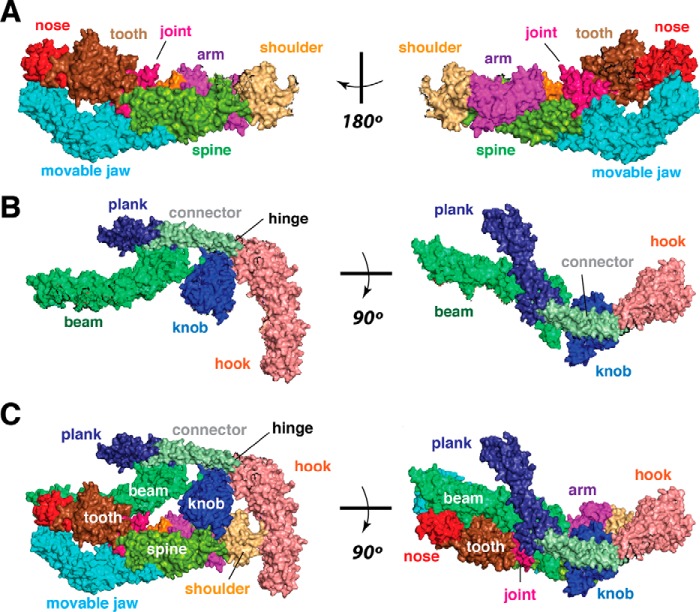
**Overview of yeast Mediator head and middle modules.** *A,* head module structural domains, based upon designations from Cramer and co-workers (11). *B,* middle module structural domains, based upon data from Asturias and co-workers (12) and Cramer and co-workers (11). *C,* two views of the middle and head modules together (*S. pombe*). Domains are colored as in *A* and *B*. Structures built from PDB 5N9J (11) using PyMOL.

The arm domain, as defined in yeast Mediator, consists of a four-helix bundle with residues from the Med6, Med8, and Med17 subunits (25). The arm resides in the head module and is adjacent to the spine and shoulder (Fig. 2*A*). The arm domain is important for binding the pol II CTD; a co-crystal structure of a 35-residue (five heptad repeats) pol II CTD and the *S. cerevisiae* head domain showed a CTD interaction surface involving α-helices within the arm domain (Med17 and Med8), as well as interactions with the adjacent shoulder domain (Med6) (20, 22, 26). These findings are consistent with subsequent structural studies with *S. pombe* Mediator (12). The spine represents another highly interconnected structural element in yeast Mediator and contains seven α-helices (25). Portions of the Med6, Med8, Med11, Med17, and Med22 subunits make up the spine; moreover, through the spine's Med6 C terminus, it interacts with a structural element called the knob (11, 12), a component of the middle module that is also implicated in pol II CTD binding (see below).

Whereas the arm and spine reside in the yeast Mediator head module, the middle module (Fig. 2*B*) contains the hook and knob domains (11, 12, 20). The hook represents a large and extensively interconnected domain located at one end of the yeast Mediator complex, opposite the tail module. The hook contains structural motifs from six different yeast Mediator subunits: Med4C (C-terminal); Med7; Med10; Med14N (N-terminal); Med19; and Med21N (11, 12). Within a minimal PIC, the Mediator hook helps form a “cradle” that likely accommodates the large TFIIH complex (20). TFIIH contains a kinase (CDK7; Kin28 in *S. cerevisiae*) that phosphorylates the pol II CTD, and CXMS data suggest that the Mediator hook (*i.e.* its Med19 subunit) may be involved in binding the pol II CTD (20, 22). Interestingly, the hook also appears to play a role in binding the CDK8 module (9, 27), which is a four-subunit complex (containing CDK8, CCNC, MED12, and MED13) that stably and reversibly associates with Mediator (7, 17, 28). CDK8 module binding to Mediator is mutually exclusive with Mediator–pol II binding (27, 29, 30), and an interaction between the hook and the Cdk8 module might occlude a Mediator–CTD interaction surface, which could serve as a means by which the Cdk8 module prevents Mediator–pol II interaction (27).

The knob consists of regions from the Med4, Med7N (N-terminal), Med14, and Med31 subunits. The knob helps connect the middle and head module (Fig. 2*C*) through Med31–Med8 and Med4–Med6 interactions (11, 12) and also appears to interact with the pol II CTD upon formation of the yeast holoenzyme (Mediator–pol II complex) (12, 22). Both yeast and human Mediator have been shown to stimulate TFIIH-dependent phosphorylation of the pol II CTD (31–34). The multiple Mediator–pol II CTD interactions, involving the arm, shoulder, hook, and knob domains, likely play a role by optimally positioning the CTD for phosphorylation by TFIIH. In *S. cerevisiae*, the knob domain subunit Med31 is also implicated in Mediator association with the TREX-2 complex, which regulates mRNA export through the nuclear pore (35). Phosphorylation of the pol II CTD (*e.g.* by TFIIH) disrupts Mediator–pol II interactions (36, 37), providing a means for pol II release and TREX-2 association. In this way, the knob domain could serve as a platform for exchange of factors involved in transcription initiation and RNA processing.

## Structural interfaces between head and middle modules

**Figure 3.**
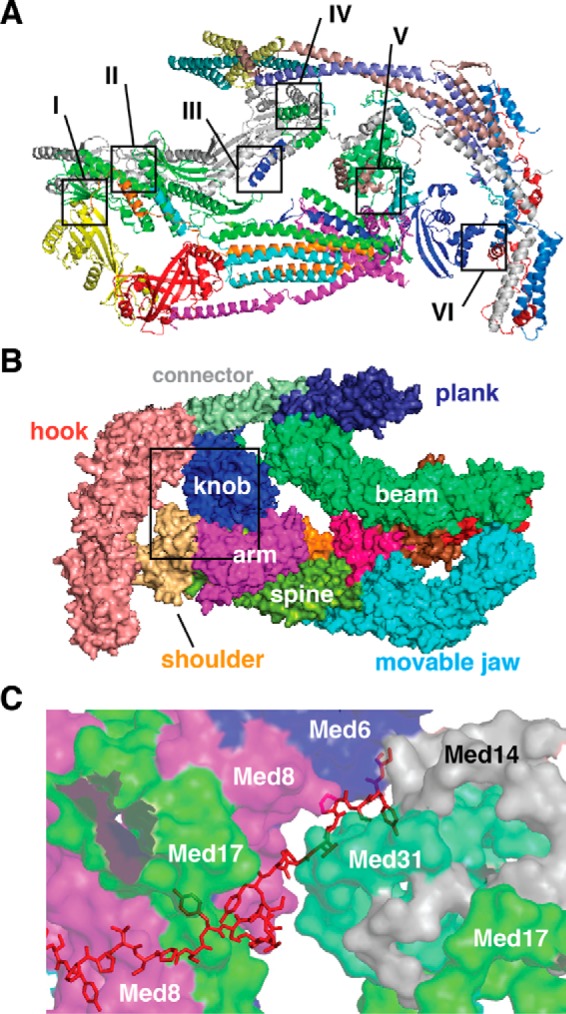
**Interfaces between yeast Mediator head and middle modules; Mediator–pol II CTD interactions.** *A,* overview of *S. pombe* Mediator head + middle modules with individual subunits shown in different colors. Head–middle interfaces are indicated with *boxed regions I–VI* as follows: *I,* Med20 (*yellow*) and Med14 (*gray*); *II,* Med17 (*green*) and Med14; *III,* C-terminal helix of Med6 (*dark blue*) and Med14; *IV,* Med17 N terminus with Med14 and Med21 (*lavender*); *V,* Med6 and Med8 (*magenta*) interaction with Med4C, Med7N, and Med31 subunits of knob domain; and *VI,* Med6 interactions with the hook domain subunits Med10 (*light blue*) and Med19 (*red*). *B, S. pombe* Mediator head + middle structure (view rotated 180° from *left panel* in Fig. 2*C*) that highlights pol II CTD interaction surface (*boxed*). Domain colors are as shown in Fig. 2*C. C*, model of the pol II CTD (*red*) and its contacts with the knob domain (Med31 and Med14 NTD) and the arm (Med17 and Med8) and shoulder (Med6) domains. The schematic in *C* reflects the structural shift that accompanies CTD–Mediator binding, as noted by Asturias and co-workers (12). Figures were generated in PyMOL, and Mediator structures are from PDB 5N9J (11) and 5U0S (12), and pol II CTD structure is from PDB 5SVA (22).

Initial high-resolution data for yeast Mediator included the head module (18, 25, 26) and a subset of middle module subunits (38, 39). Recently, however, high-resolution cryo-EM and crystal structure data have become available for *S. pombe* Mediator that have included intact head and middle modules (11, 12). These data have revealed extensive interfaces that involve Med20–Med14, Med17–Med14, Med8–Med31, Med6–Med4, Med6–Med10, and Med6–Med19, for head–middle module subunits, respectively (Fig. 3*A*). Additionally, the C terminus of Med6 and the N terminus of Med17 serve as “tethers” linking the head and middle modules, through their interactions with the Med14 backbone. Notably, the head–middle module interface appears to be flexible, and many functionally defective mutants map to head–middle protein–protein interfaces (11). This suggests that structural shifts are important for Mediator function and may involve rotation or sliding along head–middle interfaces.

## MED13, linking Mediator and the CDK8 module

Studies in both human and yeast have revealed a key role for MED13 in physically linking the CDK8 module to the Mediator complex (27, 30). Asturias and co-workers (27) provided evidence that the Cdk8 ortholog in yeast Mediator (srb10 in *S. cerevisiae*) can also directly contact Mediator, but it does not form a stable interaction on its own. Although current structural data for CDK8 module–Mediator complexes are low-resolution, the structural interface involving MED13 may be extensive, especially for the human complex (30). Based upon cryo-EM data with yeast Mediator, it appears that the Med13 interface may involve the hook domain subunits Med19 and Med14 (9, 27). CXMS data from Roeder and co-workers (13) suggest MED19 and MED14 form a similar structural domain in human Mediator. Moreover, MED14 was observed to co-purify with CDK8 modules purified from human cells (40). Collectively, these results suggest that structural aspects of CDK8 module association with Mediator may be conserved between yeast and humans, but additional data are needed for verification.

Although little is known about the structure of the MED12 or MED13 subunits of the CDK8 module (human subunits are each ∼250 kDa), crystal structures of the human CDK8–CCNC dimer have been obtained and reveal expected structural features for a cyclin-kinase dimer (41). A rough outline of the subunit organization has been obtained for the yeast Cdk8 module, which indicates a structural role for Med12 to connect the Cdk8–Ccnc dimer to Med13 (27). In agreement, biochemical experiments suggest that human MED12 is important for stabilizing the CDK8–CCNC association within the module, through physical association with CCNC (42). A MED12–CCNC interaction may explain why MED12 is required to activate human CDK8 kinase activity (40).

Association of Mediator with pol II is mutually exclusive with the CDK8 module (17, 43, 44), suggesting CDK8 module–Mediator association regulates pol II recruitment to gene promoters. Recent data from yeast support this idea and also suggest that the Cdk8 module could help form or stabilize short-range DNA upstream activating sequence (UAS) interactions with promoters in yeast (45, 46). Given the high-affinity binding of Mediator for the pol II CTD, shown for human and yeast complexes (22, 47), it is remarkable that upon Mediator–CDK8 module binding, the Mediator–pol II interaction is blocked (27, 29, 30). This suggests that the multiple pol II CTD interaction sites within Mediator become blocked, through conformational changes or physical occlusion by the CDK8 module (or both). Low-resolution structural data with human complexes suggest that conformational changes upon Mediator–CDK8 module interaction block pol II binding (17, 30). By contrast, in yeast, structural data suggest direct, physical occlusion that may involve interactions not only with Med13 but also Cdk8 (27, 29).

## Mediator interactions with RNA polymerase II

Initial low-resolution EM reconstructions of Mediator–pol II assemblies from both yeast and humans provided evidence for an extensive network of protein–protein interactions between these complexes (8, 48–50). Based upon more recent work from yeast, some of these interactions have become more clearly defined.

The structural data with yeast Mediator are generally consistent and reveal several key interfaces with the pol II enzyme. The most well-established, and likely the most important, Mediator–pol II interaction involves the CTD of the Rpb1 subunit of pol II. Using *S. cerevisiae* head module crystals soaked in CTD peptide (five heptad repeats), Kornberg and co-workers (26) were first to show structural evidence for pol II CTD interaction with Mediator subunits Med6, Med8, and Med17. These data suggested a path for the long, flexible CTD along the Mediator arm and shoulder (Fig. 3, *B* and *C*). Later, in a remarkable series of experiments, Kornberg and co-workers (22) demonstrated that yeast (*S. cerevisiae*) Mediator binds the pol II enzyme with sub-nanomolar affinity and that removal of the CTD decreased pol II binding affinity for Mediator by several orders of magnitude. This high-affinity Mediator–pol II CTD interaction appears to be conserved in human Mediator (47), although precise binding affinity measurements have not been completed with human factors.

In addition to Med6, Med8, and Med17, the knob domain (middle module in yeast Mediator) appears to interact with the pol II CTD in a structurally rearranged state induced by formation of a stable holoenzyme (*i.e.* a Mediator–pol II complex) (12, 22). Cryo-EM data from Asturias and co-workers (12) indicates a Mediator structural shift upon pol II binding. This structural shift repositions the head and middle modules and appears to trigger new Mediator interactions with the pol II CTD and the pol II foot domain. In particular, the knob domain shifts and rotates toward the spine and shoulder to facilitate interaction with the pol II CTD, which maintains interactions with Med6, Med8, and Med17 (Fig. 3*C*). Also, the Med4–Med9 “plank” domain (middle module, Fig. 2, *B* and *C*) shifts to contact the foot domain of Rpb1 (12). These structural changes can be attributed primarily to Med14, whose role as a central backbone for Mediator allows it to control relative orientations of the head and middle modules simultaneously. Biochemical and genetic data also support an interaction between the yeast Mediator knob domain and the pol II CTD. Deletion of Med31 (a knob domain subunit) is synthetic lethal with pol II CTD truncations (51) and Med31 mutation or deletion reduced Mediator–pol II association in biochemical assays (12).

Conaway and co-workers (52) provided additional evidence supporting structural changes in stabilizing Mediator–pol II interactions. Deletions or point mutations in the Mediator hinge domain (Med7C–Med21), which interacts with the knob, prevented stable Mediator–pol II association. The hinge is structurally dynamic and links the hook with a “connector” domain (Fig. 2, *B* and *C*) that connects the hook with the plank (Med4–Med9) (11). The connector contains two long α-helices, one from Med7 and one from Med21. The structural flexibility and placement of the hinge between the connector and the base of the hook may enable re-positioning of the hook, knob, and plank domains during Mediator–pol II binding (12).

Additional Mediator–pol II interactions involve the arm domain subunits Med8 and Med17, which form an interface with the pol II subunit Rpb4 (20) that, together with Rpb7, comprises the pol II stalk (53). Other Mediator head module interactions with pol II involve Med20 and the pol II subunit Rpb3, and the pol II dock domain (Rpb1) with Med18 and Med20 (12, 20). Using CXMS, Cramer and co-workers (20) and Kornberg and co-workers (22) identified cross-links between the C terminus of Med14 and the pol II subunit Rpb11. Because the Med14 C terminus connects to yeast Mediator tail module subunits (21), this result is consistent with localization of the tail along the “back” of the pol II enzyme, as shown in recent structural models (12, 20, 22).

Although it is clear that Mediator interacts extensively with the pol II enzyme, Mediator also broadly regulates the assembly and function of the entire PIC (54–57). Much remains unknown about the molecular mechanisms by which Mediator controls the structure and function of the PIC; however, key details continue to emerge (58), mainly from experiments with yeast factors. Cryo-EM analysis of a minimal yeast initial transcribing complex containing promoter DNA and a short RNA template, pol II, TBP, TFIIF, TFIIB, and a 15-subunit core Mediator complex showed evidence that Med18 interacts with the B-ribbon of TFIIB (20). Furthermore, CXMS experiments have identified cross-links between Med19 (hook domain in the middle module) and the pol II CTD (20, 22). The Mediator hook, arm, and plank (Med4–Med9) domains help form a “cradle” that appears important to accommodate TFIIH assembly within the PIC (20). This structural organization, combined with pol II CTD association with the arm, knob, and hook domains, may explain how Mediator stimulates pol II CTD phosphorylation by the TFIIH-associated kinase (yeast Kin28; human CDK7).

## Structural studies show that Mediator is a moving target

Understandably, much effort has been devoted toward finding consistency among Mediator structures studied by different labs, and the results have generally been consistent for the higher-resolution data available for yeast Mediator. However, Mediator is structurally dynamic; the tail domain of yeast Mediator remains unresolved at higher resolution due to its conformational heterogeneity (12, 21), and modest structural changes can even be observed in different crystals derived from the same purified complexes (25). Computational analyses with human and yeast Mediator subunit sequences reveal unusually high percentages of intrinsically disordered regions within its subunits (14). Asturias and co-workers (12) and Kornberg and co-workers (8) have provided evidence that yeast Mediator undergoes coordinated structural shifts upon binding the pol II enzyme, and structural shifts are also evident with the human Mediator complex upon binding pol II (47–49). Moreover, both yeast and human Mediator complexes appear to undergo structural shifts upon interaction with the activation domains of DNA-binding transcription factors (9, 17, 32, 59). Collectively, these findings reveal that Mediator structure is context-dependent and that its structural state may change upon interaction with other proteins (*e.g.* DNA-binding transcription factors or the pol II CTD). For these reasons, Mediator is a “moving target” in structural biology, which is both appealing and confounding (60).

Nogales and co-workers (61) observed that human TFIID undergoes structural rearrangements in which an ∼400-kDa subassembly (consisting of the subunits TAF1, TAF2, and TBP) can spontaneously sample two different interfaces within the TFIID complex, each separated by about 100 Å. These distinct locations for the TAF1/TAF2/TBP subassembly had functional consequences, and one specific rearranged state was stabilized upon binding the PIC factor TFIIA and promoter DNA (61). It is not known whether Mediator, a complex of similar size to TFIID, can undergo this type of structural rearrangement; however, Mediator subcomplexes that may undergo structural rearrangements similar to those observed with human TFIID include the CDK8 module and the tail module. The CDK8 module reversibly interacts with Mediator (27, 30, 62–64) and appears to associate via the flexible hook domain in yeast (27); the tail module is highly dynamic and contains the subunits Med2, Med3, Med5, Med15, and Med16 (*S. cerevisiae*). Whereas crystal structure and cryo-EM data reveal that the head and middle modules can be fairly rigid, the tail module is unusually flexible in yeast Mediator, precluding its structural characterization at higher resolution (12, 21).

Structural transitions appear to be essential for Mediator function as a regulator of pol II transcription. For example, many functionally defective or lethal mutations identified from yeast genetics experiments map to flexible domains or interfaces within Mediator (11, 25). In cells, DNA-binding transcription factors are the primary regulators of pol II activity across the genome (65). Yet, in eukaryotes, transcription factors do not directly interact with pol II but instead communicate their regulatory signals through complexes such as Mediator. Conformational changes in Mediator, induced by transcription factor binding, correlate with activation of pol II transcription *in vitro* (32). In agreement, Roeder and co-workers (13) were able to show that MED14 was required for basal and activated transcription *in vitro*. Because yeast Med14 appears to be essential to direct structural shifts needed for stable pol II association (12), these *in vitro* functional data collectively suggest that structural changes (*e.g.* mediated through MED14) are required for Mediator-dependent activation of pol II transcription. The potential for multiple distinct, functionally relevant structural states for Mediator is high, given that its conformation changes upon interaction with DNA-binding transcription factors (*e.g.* p53, SREBP, and Gcn4) and given its extensive set of interactions within the PIC. Mediator–PIC interactions are certain to change during pol II transitions from PIC assembly, initiation, and promoter escape; accurately characterizing Mediator structure during these transcription initiation stages will be an important but challenging endeavor.

## Concluding remarks

As our understanding of Mediator structure continues to expand, it will be important to link these findings to a more detailed understanding of Mediator function and mechanism. Studies with yeast Mediator can draw upon extensive genetic data that have linked mutations or deletions to specific functional defects *in vivo*. In parallel, clinical data continue to link human Mediator subunits to specific types of cancer or developmental diseases. Whereas these types of data can help validate key functional roles, it is equally important that structure–function relationships are tested further, not only with cell-based and *in vivo* studies but also with *in vitro* experiments. *In vitro* studies should be especially informative because mutations that affect key structural interfaces in Mediator are likely to yield cell lethal phenotypes whose functional roles might only be reliably assessed with *in vitro* assays. Moreover, because of the high level of experimental control (*e.g.* factor titration, rapid timing, and order of addition), *in vitro* assays can better assess precise molecular mechanisms that can be difficult, if not impossible, to determine with cell-based techniques. Given the innovations in cryo-EM data collection and image processing (66–68), combined with advances in structural proteomics techniques (69, 70), it is an exciting time for structural biology. Biophysical and functional analysis of Mediator in different contexts will continue to yield important mechanistic insights that should be increasingly relevant for human disease and the development of molecular therapeutics (71–75).

## Footnotes

2 The abbreviations used are:

pol: polymerase
PIC: preinitiation complex
CXMS: cross-linking mass spectrometry
CTD: C-terminal domain
PDB: Protein Data Bank.
